# Transcriptome Analyses of Myometrium from Fibroid Patients Reveals Phenotypic Differences Compared to Non-Diseased Myometrium

**DOI:** 10.3390/ijms22073618

**Published:** 2021-03-31

**Authors:** Emmanuel N. Paul, Gregory W. Burns, Tyler J. Carpenter, Joshua A. Grey, Asgerally T. Fazleabas, Jose M. Teixeira

**Affiliations:** Department of Obstetrics, Gynecology and Reproductive Biology, Michigan State University College of Human Medicine, Grand Rapids, MI 49503, USA; paulemma@msu.edu (E.N.P.); burnsgr2@msu.edu (G.W.B.); carpe290@msu.edu (T.J.C.); greyjosh@msu.edu (J.A.G.); fazleaba@msu.edu (A.T.F.)

**Keywords:** uterine fibroids, myometrium, field effect, CCND1, SERPINE1

## Abstract

Uterine fibroid tissues are often compared to their matched myometrium in an effort to understand their pathophysiology, but it is not clear whether the myometria of uterine fibroid patients represent truly non-disease control tissues. We analyzed the transcriptomes of myometrial samples from non-fibroid patients (M) and compared them with fibroid (F) and matched myometrial (MF) samples to determine whether there is a phenotypic difference between fibroid and non-fibroid myometria. Multidimensional scaling plots revealed that M samples clustered separately from both MF and F samples. A total of 1169 differentially expressed genes (DEGs) (false discovery rate < 0.05) were observed in the MF comparison with M. Overrepresented Gene Ontology terms showed a high concordance of upregulated gene sets in MF compared to M, particularly extracellular matrix and structure organization. Gene set enrichment analyses showed that the leading-edge genes from the TGFβ signaling and inflammatory response gene sets were significantly enriched in MF. Overall comparison of the three tissues by three-dimensional principal component analyses showed that M, MF, and F samples clustered separately from each other and that a total of 732 DEGs from F vs. M were not found in the F vs. MF, which are likely understudied in the pathogenesis of uterine fibroids and could be key genes for future investigation. These results suggest that the transcriptome of fibroid-associated myometrium is different from that of non-diseased myometrium and that fibroid studies should consider using both matched myometrium and non-diseased myometrium as controls.

## 1. Introduction

Uterine fibroids, also known as uterine leiomyomas, are benign monoclonal steroid-dependent tumors of the smooth muscle compartment (myometrium) of the uterus [1,2]. Although benign, uterine fibroids are the most common reproductive tract tumors in reproductive age women, with an incidence up to 80% [3,4], depending on race and ethnicity [5]. The most common reasons women with symptomatic fibroids seek medical attention are heavy and prolonged menstrual bleeding, anemia, fatigue, pelvic pain, obstruction of the surrounding pelvic structures induced by large fibroids, dysmenorrhea, urinary incontinence, constipation, lower back pain, sexual dysfunction, infertility and recurrent pregnancy loss [6]. Hysterectomy, the most common and effective treatment for uterine fibroids, results in permanent infertility. Despite the significant healthcare burden posed by uterine fibroids and their negative impact on the quality of life of many women, the etiology and pathogenesis of the disease are not well understood. This gap in knowledge has likely been the major reason that effective, long-term and fertility-sparing clinical management of the disease has been elusive.

Uterine fibroids can be divided into two major subtypes based on genetic alterations. Genome wide exome sequencing showed that between 50–70% of fibroids, depending on patient ethnicity and fibroid number, contained mutations primarily in the second exon of the Mediator Complex subunit 12 (*MED12*) gene [7]. These are the *MED12*mt fibroid subtype. Chromosomal rearrangements at 12q14–15 near the *HMGA2* gene, which result in *HMGA2* overexpression, have been observed in 8–35% of fibroids and represent the *HMGA2*hi subtype [8,9]. *HMGA2* is thought to be an oncogene that is not normally expressed in differentiated adult tissues [10]. Together, the *MED12*mt and *HMGA2*hi fibroid subtypes represent the genetic alterations found in the vast majority of fibroids.

Transcriptomic analysis of fibroids has been used to understand their pathogenesis and has helped with the discovery of several dysregulated genes in the tissue [11,12]. However, to our knowledge most fibroid studies have compared fibroid tissues with the matched ‘normal’ myometrial tissues from the same hysterectomy, and little is known about the transcriptomic profile of adjacent myometrium and how it is influenced by the fibroid –the so-called field effect [13]. Myometrial tissue collected from the same resected uterus as fibroids has been considered healthy tissue since its histology appears normal, under the assumption that histological normalcy implies biological normalcy [14]. Although the pairwise study design of fibroids versus matched myometrium has proven valuable to detect differences between the tumor and the adjacent tissue [15], we hypothesize that, even if myometrial tissues from fibroid patients appear histologically normal, the transcriptomic profile or phenotype of the tissue is different from non-fibroid/non-diseased myometrium. If indeed the matched myometrium is likely not entirely normal, comparing these matched tissues could miss important genes involved in the pathogenesis or early etiology of the disease. For example, gene expression differences have been observed in histologically normal breast epithelium of breast cancer patients when compared to that of cancer-free patients [16]. Moreover, the adjacent tissue of different cancer tumors presented a unique intermediate state between healthy and tumor [14]. To test our hypothesis, we compared the transcriptome of myometrial samples from non-fibroid patients, with samples from fibroids with the most common fibroid subtype, *MED12*mt [17], and their matching “normal” myometrium and showed that, although the two tissues are clearly more alike than not when compared to fibroid tissues, there is a distinct phenotype for each.

## 2. Results

### 2.1. Myometrial Samples from Fibroid Patients Differentially Express Fibroid-Associated Genes

**Figure 1:**
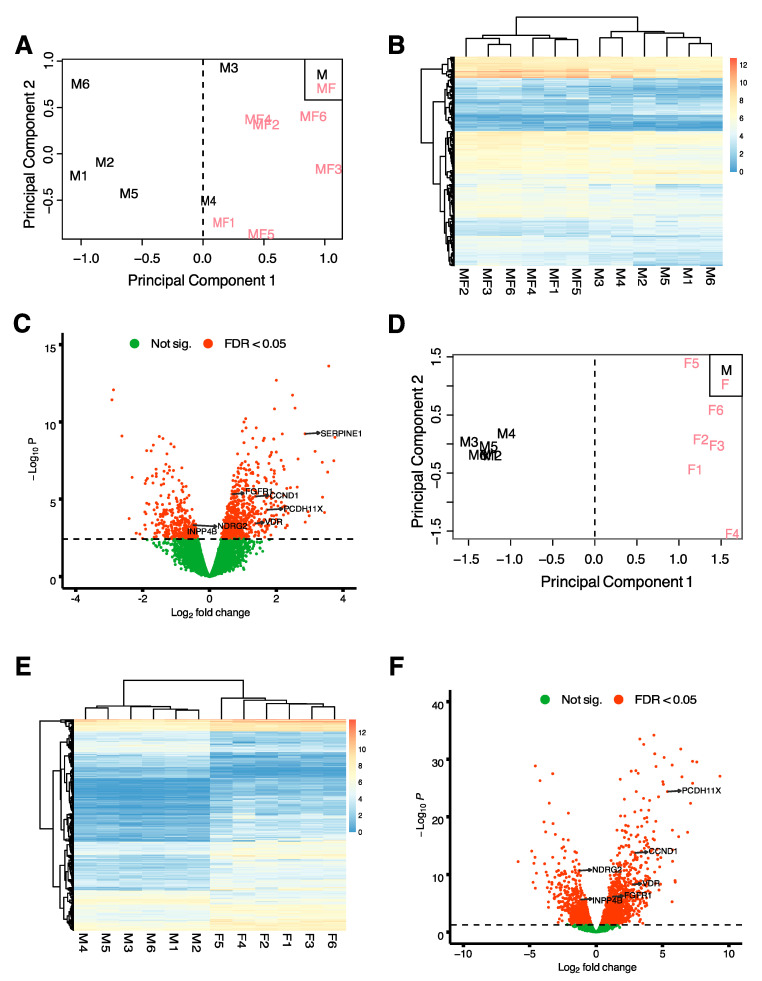
Comparison of transcriptome profiles of myometrial samples from non-fibroid patients (M) with myometrial (MF) and fibroid (F) samples from fibroid patients. (**A**) Multidimension scaling (MDS) plot of RNA-seq results from non-fibroid myometrial samples compared with samples from fibroid-bearing myometria. Each label represents one sample. Myometrial samples from non-fibroid patients (M) are shown with black letters (*n* = 6) and myometrial samples from fibroid patients (MF) are shown with red letters (*n* = 6). (**B**) Heatmap of the top 500 differentially expressed genes (DEGs) from the MF vs. M comparison with unsupervised hierarchical clustering of genes and samples (*n* = 6/group). (**C**) Volcano plot showing up (*n*= 705) and downregulated genes (*n* = 464) with a false discovery rate (FDR) *p*-value < 0.05 in MF vs. M depicted as red dots. (**D**) MDS plot of RNA-seq results from non-fibroid myometrial samples compared with fibroid samples. Each label represents one sample. Myometrial samples from non-fibroid patients (M) are shown with black letters (*n* = 6) and fibroid samples (F) are shown with red letters (*n* = 6). (**E**) Heatmap of the top 500 DEGs from the M vs. F comparison with unsupervised hierarchical clustering of genes and samples (*n* = 6/group). (**F**) Volcano plot showing up (*n* = 1736) and downregulated genes (*n* = 1323) with FDR *p*-value < 0.05 in M vs. F depicted as red dots. Color gradient represents gene expression as log_2_(CPM + 1) in B and E.

To determine whether the transcriptomic profiles of tissue samples from myometria of non-fibroid patients (M) and from myometria of *MED12*mt fibroid patients (MF), are comparable and/or distinct from each other and uterine fibroids, we performed RNA-seq analysis of tissue samples from M (*n* = 6), MF (*n* = 6) and *MED12*-mutant fibroids (F) (*n* = 6). Multidimensional scaling (MDS) plots were generated to visualize the pairwise differences between samples of MF vs. M (Figure 1A). The MDS plot of MF vs. M showed that the MF samples were separated from the M samples by principal component (PC) 1, indicating gene expression differences between the MF and M groups. A total of 1169 genes were differentially expressed (DE) with a false discovery rate (FDR) < 0.05, including 464 decreased and 705 increased, in the MF comparison with M (Supplementary file 1). A heatmap, performed by unsupervised hierarchical clustering of the top 500 DE genes (DEGs) between the samples, confirmed that samples grouped by tissue type (Figure 1B). A volcano plot was used to visualize the distribution of DEGs (Figure 1C). Genes of interest involved in tumor progression or uterine fibroids, from previous studies, *FGFR1* [18], *CCND1* [19], *PCDH11X* [20], *VDR* [21], *NDRG2* [22] and *INPP4B* [23], are indicated in the volcano plot. *NDRG2* and *INPP4B* are significantly downregulated in MF compared to M with a log_2_ fold change (FC) of −0.5 and −0.6, respectively. *FGFR1*, *CCND1*, *PCDH11X*, and *VDR* are significantly upregulated in MF compared to M, with a log_2_FC of 0.7, 1.4, 1.7 and 1.4, respectively. These results support downregulation of tumor suppressor genes and upregulation of pro-tumoral genes within the myometria of *MED12*mt fibroids, compared to non-fibroid myometria.

We also compared transcriptomic profiles of F vs. M. The MDS plot shows distinct grouping by tissue type (Figure 1D), with M samples being more separate from the F samples by PC 1 compared to the MF samples (Figure 1A), as indicated by the X axis. This relationship was confirmed by a larger difference in gene expression between F and M compared to MF and M tissue types. Overall, 3059 DEGs were found between F vs. M, with 1323 downregulated genes and 1736 upregulated genes (Supplementary File 2). A heatmap showing unsupervised hierarchical clustering of the top 500 DEGs showed greater gene expression fold changes in F vs. M (Figure 1E) when compared to the MF vs. M heatmap (Figure 1B). A higher number of DEGs with a fold change >2 (1613 total genes) was observed in the F vs. M comparison (Figure 1F) compared to the MF vs. M comparison (494 total genes, Figure 1C) as illustrated by the volcano plots. Additionally, the log_2_FC of the DEGs of interest described above were greater in the F comparison. *NDRG2* and *INPP4B* were downregulated with a log_2_FC of −1.3 and −1.2, respectively. *FGFR1*, *CCND1*, *PCDH11X*, and *VDR* were upregulated with a log_2_FC of 1.2, 2.9, 5.4 and 2.7, respectively.

### 2.2. Myometrial Samples from Fibroid Patients Are Enriched for Multiple Gene Sets That May Be Involved in the Development of the Disease

**Figure 2:**
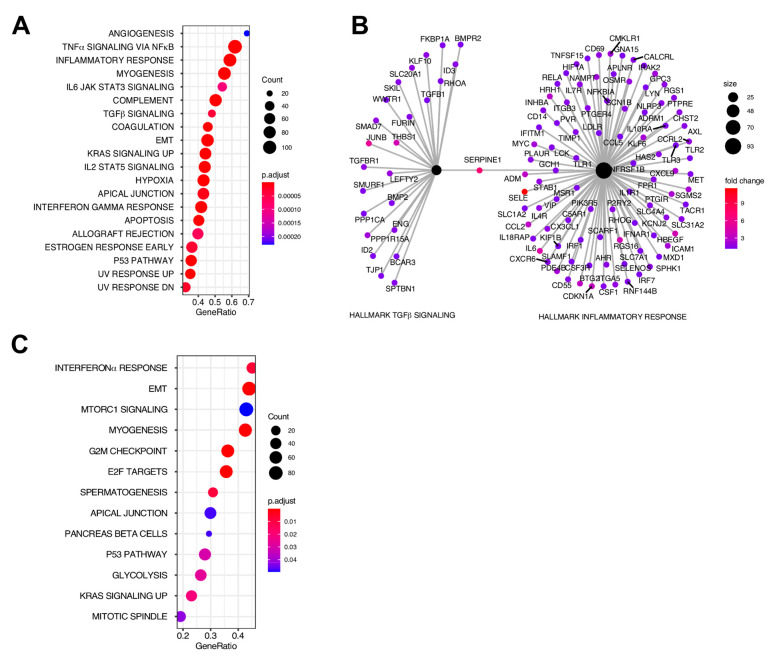
Gene set enrichment analysis (GSEA) of myometrial samples from non-fibroid patients (M) compared with myometrial samples from fibroid patients (MF) and with fibroids (F). (**A**) Top significantly enriched gene sets from the MF vs. M by GSEA using Hallmark biological processes in MSigDB. (**B**) Cnetplot of TGFβ signaling and inflammatory response processes enriched gene sets in MF vs. M. *SERPINE1* was in each set and is shown here connecting the two nodes. Fold change and number of genes in each node are indicated by the color gradient and circle size, respectively. (**C**) Significantly enriched gene sets (F vs. M) from GSEA using Hallmark biological processes in MSigDB. Gene count and significance level are shown by the size and color of each circle, respectively, in A and C.

To determine whether the DEGs discovered above affect the myometrial phenotype, we analyzed the top enriched Hallmark-curated gene sets [24] when comparing MF vs. M and F vs. M by gene set enrichment analysis (GSEA). A total of 20 gene sets with a significant adjusted *p*-value < 0.05 were enriched and activated in MF vs. M (Figure 2A). Notably, these included TGFβ signaling and multiple inflammatory Hallmark gene sets associated with tumorigenesis. In addition, when plotting the leading-edge genes from the TGFβ signaling and inflammatory response gene sets, a network plot [25] showed *SERPINE1*, which was highly upregulated in MF compared to M (Figure 1E), connected both of the Hallmark gene sets (Figure 2B). There were 13 significantly enriched Hallmark gene sets by GSEA when F was compared to M (Figure 2C), which is fewer than the 20 observed when comparing MF vs. M.

**Figure 3:**
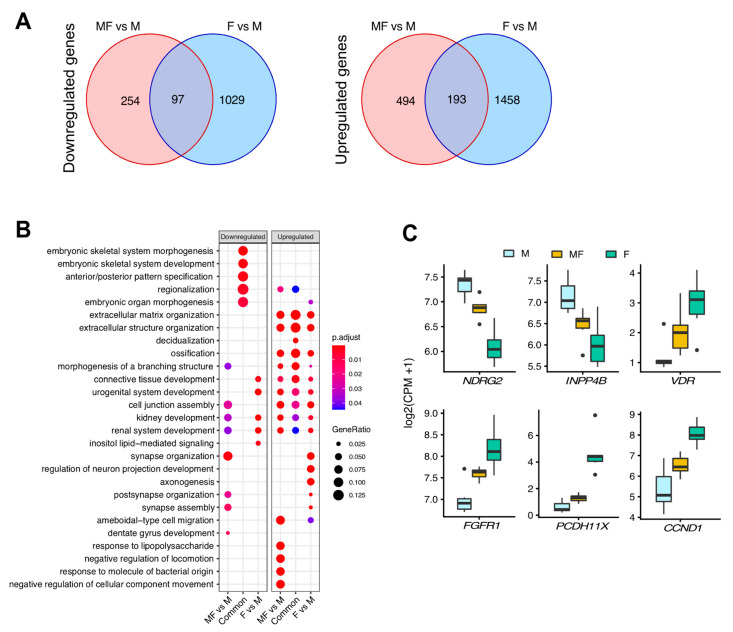
Up and downregulated overlapping DEGs in myometrial samples from fibroid patients vs myometrial samples from non-fibroid patients (MF vs. M) and fibroids vs myometrial samples from non-fibroid patients (F vs. M). Venn diagrams illustrate the overlap of the down (**A**) and upregulated genes between MF vs. M and F vs. M. (**B**) Top overrepresented Gene Ontology pathways analyses for MF vs. M-specific, shared, and F vs. M-specific up and downregulated gene sets. Gene enrichment ratio and significance levels are shown by the size and color of each circle, respectively. (**C**)Boxplot of significant up or downregulated genes (false discovery rate *p*-value < 0.05 and fold change > 2) associated with fibroids and tumorigenesis (*n* = 6/group). Gene expression is shown as log_2_(CPM + 1).

To investigate whether the transcriptomic changes observed in MF vs. M samples suggest that MF tissue has a transitional phenotype between M and F tissues, the DEGs of MF vs. M were overlapped with the DEGs from F vs. M (Figure 3A). A total of 97 downregulated and 193 upregulated DEGs overlapped between MF vs. M and F vs. M. The unique downregulated and upregulated, and overlapping DEGs were analyzed by over-representation analysis using biological process Gene Ontology (GO) terms. The overrepresented GO terms showed a high concordance of upregulated gene sets, particularly with the extracellular matrix organization and extracellular structure organization (Figure 3B), pathways known to be upregulated in uterine fibroid disease. The log_2_(CPM + 1) of dysregulated genes highlighted in Figure 1C and F (*FGFR1*, *CCND1*, *PCDH11X*, *VDR*, *NDRG2* and *INPP4B*) are shown in boxplots grouped by tissue type (Figure 3C). The plots show a progression of gene expression from M to MF to F, in agreement with the previous results and supporting a transitional phenotype for myometria from fibroid patients.

### 2.3. Overall Comparison of Myometrial Samples from Non-Fibroid Patients (M), Myometrial Samples from Fibroid Patients (MF), and Fibroids Samples (F)

**Figure 4:**
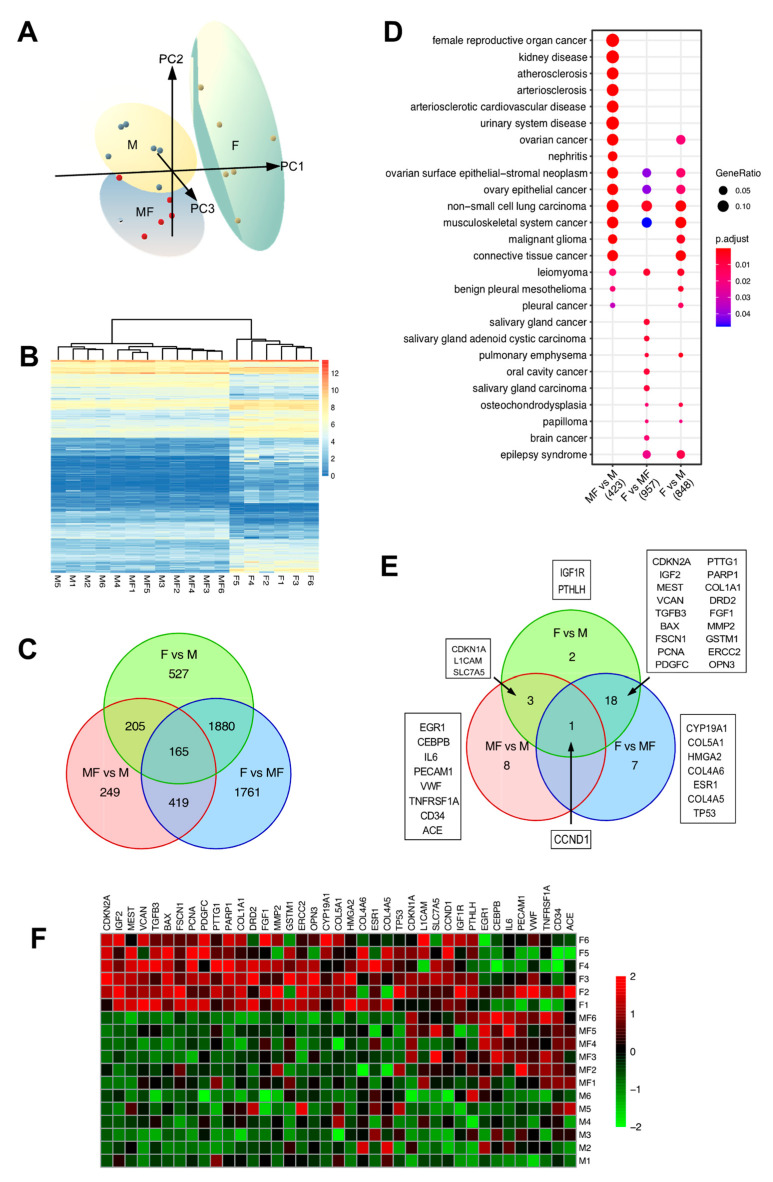
Transcriptome characterization of all 3 groups: myometrium from non-fibroid patients (M), myometrium from fibroid patients (MF) and fibroids (F) (**A**) 3D Principal component analysis plot based on RNA-seq gene expression data. Ellipses represent 95% confidence in each group (*n* = 6/group). Yellow, blue and green ellipses represent the group M, MF and F, respectively. Individual dots represent each sample analyzed. (**B**) Heatmap of the top 500 differentially expressed genes (DEGs)of M, MF, F groups with unsupervised hierarchical clustering of genes and samples (*n* = 6/group). Color gradient represents gene expression levels as log_2_(CPM + 1). (**C**) Venn diagram illustrates the DEG overlapping between MF vs. M, F vs. MF, and F vs. M (5206 genes total). (**D**) Top overrepresented Disease Ontology terms for MF vs. M, F vs. MF, and F vs. M upregulated genes. Gene enrichment ratio and significance level are shown by the size and color of each circle, respectively. (**E**) Venn diagram leiomyoma disease ontology genes illustrates the overlap between MF vs. M, F vs. MF, and F vs. M (39 genes total). (**F**) Heatmap of the 39 leiomyoma enriched genes in MF vs. M, F vs. MF, and F vs. M (*n* = 6/group). Color gradient represents gene expression levels as z-scores.

A 3D principal component plot generated using all samples and expressed genes shows M and MF samples are separated from fibroid samples by PC 1 (Figure 4A, PC1 = 21.8% variance). In contrast, M and MF samples were separated by PC2, indicating that overall gene expression is more similar between these tissue types (PC2 = 15.9% variance). These inferred distances were confirmed by unsupervised hierarchical clustering of the samples using the top 500 DEGs, as illustrated in a heatmap dendrogram (Figure 4B). DEGs from pairwise comparisons of MF vs. M (*n* = 1038), F vs. MF (*n* = 4625), and F vs. M (*n* = 2777), showed overlapping DEGs between all groups (Figure 4C). To determine the strength of the F vs. MF results, we compared the current list of DEGs between *MED12*mt fibroids and matching myometria with those discovered in our previous study [11]. The comparison showed a significant overlap between studies (hypergeometric test *p* < 0.0001), with 97.8% of the previously reported downregulated DEGs and 97.2% of upregulated DEGs included in the F vs. MF analysis (Figure S1). The majority of DEGs from F vs. M overlapped with F vs. MF (*n* = 2045, 73%). Interestingly, 76% of the DEGs from MF vs. M (789/1038) were common between the two fibroids comparisons (F vs. M and F vs. MF), another indication that uterine fibroid related genes are altered in MF tissues. Importantly, 732 DEGs from F vs. M were not found in the F vs. MF and may be understudied genes involved in the pathogenesis of leiomyomas.

**Figure 5:**
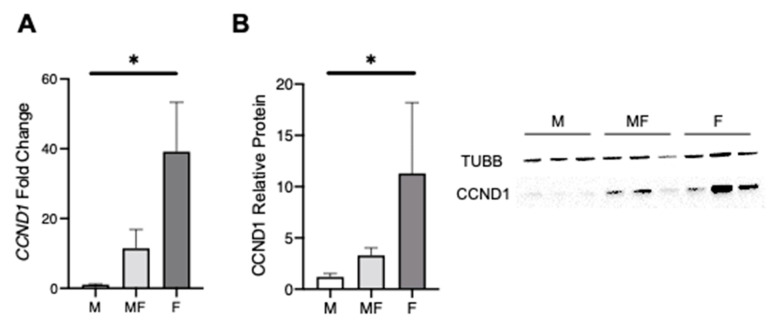
*CCND1* gene and protein expression in myometria from non-fibroid patient (M), fibroid (F) and matching myometria (MF). (**A**) Relative expression of *CCND1* by qRT-PCR compared with the RPL17 housekeeping gene in M, MF and F (*n* = 5–6/group), * *p*-value = 0.01. (**B**) Representative western blot and quantification of the relative protein expression of CCND1 normalized to TUBB in M, MF and F tissues (*n* = 6/group), * *p*-value = 0.01.

To investigate which of the DEGs have a reported role in uterine fibroids, Disease Ontology Semantic and Enrichment (DOSE) analysis [25] was performed (Figure 4D). Most of the disease gene sets, including leiomyoma, were enriched in F vs. MF and in F vs. M comparisons (FDR *p*-value < 0.007 and 0.005, respectively). Notably, MF vs. M was enriched for the leiomyoma gene set (FDR *p*-value < 0.016) along with F vs. MF and F vs. M comparisons. The DEGs from the leiomyoma gene set were visualized using a Venn diagram, a heatmap and boxplots (Figure 4E,F and Figure S2). As expected, most of the leiomyoma enriched genes were found in both F vs. MF (*n* = 26) and F vs. M (*n* = 24) tissue types with 19 overlapping genes. A total of 13 leiomyoma genes were found in F vs. M and MF vs. M, but not in F vs. MF, indicating that the more common F vs. MF comparison could miss important genes involved in uterine fibroid development. Most of the leiomyoma DEGs were found upregulated in F compared to the MF or M (Figure 4F and Figure S2); however, some genes, including *CDKN1A*, *L1CAM*, *SLC7A5*, *CCND1*, *IGF1R*, and *PTHLH*, were only increased in MF vs. M and F vs. M comparisons (Figure 4E and Figure S2). *CCND1*, or Cyclin D1, is a DEG in all three comparisons that is increased in F (compared to M and MF), as well as in MF compared to M (Figure 4F and Figure S2). We validated the *CCND1* results with tissues from a different set of patients (*n* = 5–6) by qPCR and Western blot analyses (Figure 5). *CCND1* relative expression was significantly higher in F compared to MF and M, and also increased in MF compared to M (Figure 5A). Cyclin D1 protein expression was also increased in MF and F when compared to M (Figure 5B), supporting the results from RNA-seq analysis. Since *CCND1* was the only overlapping gene in all three comparisons, we investigated upstream pathways of CCND1, including the WNT signaling pathway, components of which have been shown to be upregulated in fibroid tissues [26,27,28]. Transcriptomic analysis found overrepresentation of the WNT pathway in F vs. MF only (FDR *p*-value = 0.003) (Figure S3), but the mitogen-activated protein kinase (MAPK) pathway, which is also upstream of CCND1, was upregulated in F and MF tissues when compared to M (FDR *p*-value < 0.01) (Figure S4). A total of eight leiomyoma-related DEGs (*EGR1*, *CEBPB*, *IL6*, *PECAM1*, *VWF*, *TNFRSF1A*, *CD34* and *ACE*) were found upregulated in MF versus M only (Figure 4E,F and Figure S2). These genes are associated with inflammatory response and endothelial markers and may be involved in the early pathogenesis of the fibroids.

### 2.4. Leiomyoma Gene List Involved in Early Pathogenesis and Establish Disease

**Figure 6:**
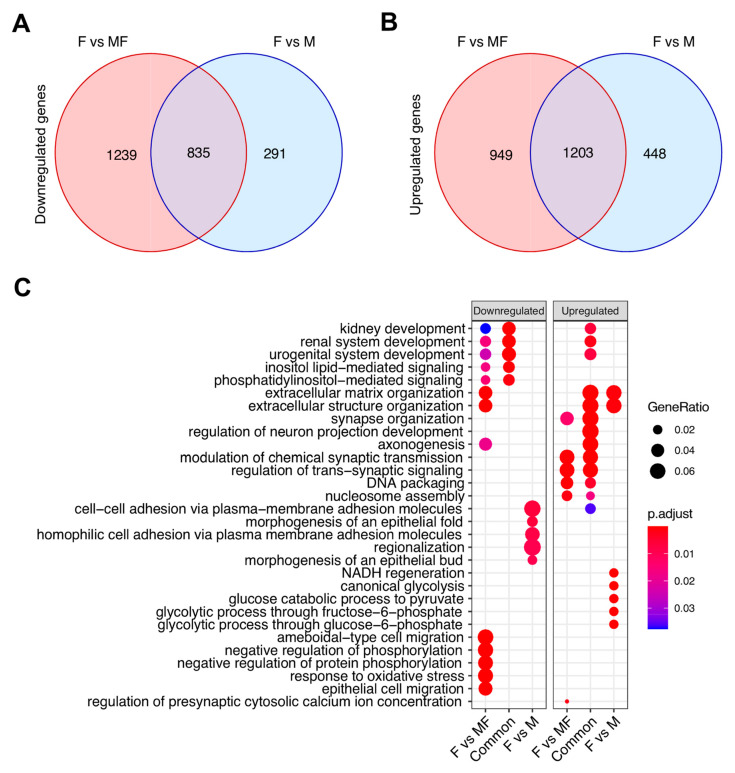
Up and downregulated overlapping genes in fibroid (F) versus matching myometrium (MF) and fibroids (F) versus myometrium from non-fibroid patients (M) Venn diagrams illustrate the overlap for downregulated (**A**) and upregulated genes (**B**) between F vs. MF and F vs. M. (**C**) Top overrepresented Gene Ontology pathways analyses for F vs. MF-specific, shared, and F vs. M-specific up and downregulated genes. Gene enrichment ratio and significance level are shown by the size and color of each circle, respectively.

Fibroid studies are commonly designed to compare fibroids to matching patient myometrium (MF) to gain understanding of the pathophysiology of the disease. Many overlapping DEGs from downregulated (Figure 6A) or upregulated (Figure 6B) F vs. M and F vs. MF comparisons were observed. However, a total of 739 DEGs were unique to F vs. M (291 downregulated + 448 upregulated DEGs). In order to determine if these genes may be important for disease progression, overrepresentation of GO biological process terms was performed in the unique DEGs of F vs. MF, F vs. M, and the common DEGs (Figure 6C). Surprisingly, extracellular matrix and structure organization gene sets, known to be involved in the leiomyoma phenotype, were overrepresented in downregulated genes unique to the F vs. MF comparison. In contrast, these gene sets were overrepresented in the upregulated genes from F vs. M and common (F vs. MF and F vs. M) DEGs. Although the enriched downregulated genes from F vs. MF (*n* = 49) did not overlap with the upregulated genes from F vs. M (*n* = 24), these results suggest that extracellular matrix structure and organization may be upregulated early in the progression of the disease, as in MF samples, and decrease in well-established tumors. In fact, 41% (20/49) of the downregulated genes in F vs. MF were increased in MF vs. M (Figure 3B).

## 3. Discussion

To our knowledge, this is the first study describing the expression of leiomyoma markers in myometria from fibroid patients and DEGs when compared to non-fibroid myometria. Our results suggest that myometrium from fibroid patients is in an intermediate stage between non-fibroid myometrium and fibroid tumors. Similar observations have been published in cancer studies, particularly in breast cancer, since healthy tissue samples can be obtained from elective reduction mammoplasty [15]. In fact, tumors may influence the molecular signatures in the genome, epigenome, transcriptome, proteome, metabolome and interactome on the surrounding tissue, a phenomenon referred to as the field effect [13]. Unlike breast cancer studies, our non-fibroid patient myometrium samples were mostly collected from hysterectomies of endometriosis patients. As such, the endometrium and adjacent underlying myometrium were removed during processing, and healthy myometrium was collected for downstream experiments to avoid potential confounding results.

A total of 1169 genes were differentially expressed in M compared to MF, including fibroblast growth factor type I receptor (*FGFR1*), cyclin D1 (*CCND1*), protocadherin 11 X-linked (*PCDH11X*), vitamin D receptor (*VDR*), N-Myc downstream-regulated gene 2 Protein (*NDRG2*) and inositol polyphosphate-4-phosphatase type II B (*INPP4B*). These genes appeared to be more dysregulated in fibroids, suggesting a possible role in the pathogenesis of the tumor. FGFR1 protein expression was reported to be increased in uterine fibroids compared to the myometrium [18,29], and its expression appears to be correlated with the size of the tumor [18]. In our study, *FGFR1* was increased in F vs. M and F vs. MF (FC = 2.30 and 1.49, respectively) but also between the two types of myometrium, MF vs. M (FC = 1.59). Moreover, the FGF signaling cascade leads to MAPK activation and increased extracellular matrix organization, pathways enriched in the fibroids and myometrium from fibroid patients in our GO analysis. This gene could be involved in the pathogenesis of the leiomyoma disease. Indeed, the FGF family, including FGFR1, are known to be involved in tumorigenesis [18,30]. Moreover, *FGFR1* upregulation has been associated with overexpression of *CCND1* [31]. CCND1, an important regulator of cell cycle progression and proliferation, is known to drive tumorigenesis [32] and was also found upregulated in uterine fibroids [33,34]. We believe that CCND1 plays a central role in disease progression since it was the only overlapping DEG found increased in all three comparisons (MF vs. M, F vs. M, and F vs. MF). CCDN1 is also a β-catenin target gene, a transcriptional factor whose expression has been shown to be upregulated in fibroids [26,27,28], and constitutive expression of an active form of β-catenin in mouse Mullerian duct mesenchyme induces fibroid-like fibrosis as the mice age [35]. Our pathway analysis showed an overrepresentation of WNT pathway in F vs. MF only, suggesting that this pathway may be dysregulated during late stages of the disease and does not likely play a role in *CCND1* over-expression of MF tissues. The MAPK pathway, also upstream of CCND1, was up regulated in MF and F, indicating a possible role in *CCND1* mRNA expression. The MAPK pathway has been previously reported to play a role in the growth of uterine fibroids [29] and should be investigated further to improve our understanding the pathogenesis of the disease. Even though, mRNA expression by qPCR or Western blot only showed an increase in F compared to M but not in MF vs. M or F vs. MF, targeting CCND1 in early disease may offer a useful avenue for therapeutic treatments. Indeed, it has been recently found that microRNA 93 (miR-93) blocks cell cycle progression and promotes apoptosis in uterine fibroid cells by targeting *CCND1* mRNA [36].

*PCDH11X*, an X-linked protocadherin gene involved in segmental development, was found upregulated in F vs. M and MF vs. M. The role of *PCDH11X* in leiomyomas is unclear, but hypomethylation of this gene has been reported in fibroids compared to the adjacent myometrium [20], which could lead to its overexpression. Surprisingly, VDR was found upregulated in F, and also in MF compared to M in our study. Since African American women are at higher risk for both developing symptomatic uterine fibroids and vitamin D deficiency, several studies have focused on the role of vitamin D in fibroids and have noted that vitamin D deficiency could be an important risk factor for uterine fibroids [37,38,39,40,41,42]. A recent report showed that combination therapy of Relugolix and vitamin D improves outcomes for women with uterine fibroid symptoms, which could lead to fewer hysterectomies for women with clinically significant fibroids [43]. Many of these studies showed that expression of VDR is lower in fibroids. In agreement with our results, VDR was upregulated in the center of the fibroids [21] and also found upregulated in *MED12*mt fibroids in our previous study [11]. Clearly, the roles of VDR and vitamin D, itself, in fibroid biology need further investigation to account for these discrepancies. Furthermore, one cannot rule out posttranslational mechanisms for VDR activation and response to vitamin D [42]. *NDRG2* and *INPP4B* are tumor suppressor genes that were downregulated in MF vs. M and lower in F vs. MF. Their role in uterine fibroids is currently unknown, but may be relevant since they were both differentially expressed in MF compare to M. Further investigation is needed to determine a role for tumor suppressors in the transformation of normal myometrium into fibroids.

Several gene sets, including TGF-β and inflammatory response, known to be upregulated in fibroids, were found enriched in MF compared to M, suggesting that MF presents some fibroid-like transcriptomic signatures [44,45]. Network pathway analysis identified *SERPINE1*, which is highly upregulated in MF compared to M (fold change 7.26, FDR = 6 × 10^−7^), as a leading-edge gene involved in both pathways. Conversely, *SERPINE1* was downregulated in F vs. MF (fold change −3.75, FDR = 5 × 10^−8^), in agreement with a previous study comparing fibroids to matching myometrium [46]. *SERPINE1* may have a role in the establishment of the disease. Indeed, *SERPINE1* has been reported to be involved in angiogenesis, observed as enriched in MF tissues compared to M, and tumorigenesis [47]. Based on this, *SERPINE1* expression in the myometrium could be used for early diagnosis of the disease and as a therapeutic target. Surprisingly, few classically associated fibroid pathways were enriched in the F vs. M comparison. The GSEA method relies on gene rank; thus, the lack of enrichment may be due to dysregulation of the fibroid transcriptome. Alternatively, this suggests that MF samples may not be the most appropriate control to identify dysregulated pathways in uterine fibroids. A list of 739 genes that were not included in the F vs. MF DEGs, but found in the F vs. M comparison (Figure 6), may contain key genes for future investigations.

As expected, F and MF tissues were enriched for fibroid gene sets. Indeed, there were 12 genes from the Disease Ontology Leiomyoma gene set that were increased in MF samples compared to M. This result is in agreement with the analysis indicating that MF samples differentially expressed some fibroid-like genes and could, therefore, represent a transition between M and F. We also noted that the list of dysregulated genes from the Leiomyoma gene set was incomplete when comparing fibroids to matching myometrium. In fact, 13 genes were not identified in the F vs. MF comparison, but were found in F vs. M and MF vs. M. Eight genes were found only in MF vs. M, suggesting that these gene may contribute to the disease establishment. Cancer studies have reported that tumor-adjacent tissue is not ‘normal’ tissue and, instead, represented a unique intermediate between healthy and tumor states [14]. One limitation of our study is the possible effect of patient somatic mutations on the gene expression. In cancer, most somatic mutations are likely to be passenger mutations; however, some mutations were found to be correlated with gene expression changes and operative in human cancer [48]. We have previously reported that the exomic mutational landscape of fibroids is relatively low compared to cancer [11], but further investigation will have to be done to determine if somatic mutations outside the exome could be correlated with the gene expression changes found in our study.

Another limitation of our study could involve variation in hormonal response of the female reproductive tract leading to a shift in RNA expression. The endometrium, in particular, is dynamically responsive to ovarian steroid hormones [49]. However, the myometrium is considered a mostly quiescent tissue, except during pregnancy, and transcriptome responses to steroid hormones during the menstrual cycle may be low. Epidermal growth factor receptor mRNA was shown to be upregulated in proliferative phase myometrium, suggesting some transcriptional response during the menstrual cycle [50]. Overall, the transcriptional response of the myometrium to cycling ovarian steroid hormones remains largely unknown. The present study used samples from both proliferative and secretory phases, as determined by a pathologist, and are randomly distributed in the tissue groups. Although this design should prevent an effect of cycle on the results, phase-matched tissues should be considered for future studies.

In conclusion, the present study demonstrates that the transcriptome of the myometrium from fibroid patients differs from non-fibroid myometrium and expresses fibroid-associated markers, suggesting that the myometrium from fibroid patients may be a transition state between the non-fibroid myometrium and the fibroid tumors. These results also support the hypothesis that myometrium from fibroid patients expresses early signs of fibroid disease that may be useful as therapeutic and diagnostic targets.

## 4. Materials and Methods

### 4.1. Sample Collection

The use of human tissue specimens was approved by the Spectrum Health Systems Institutional Review Board (MSU IRB Study ID: STUDY00002244, SR IRB #2017-198) as secondary use of biobank materials. Myometrial samples from non-fibroid patients (M), and samples from fibroids (F) and matched myometria (MF) were obtained following total hysterectomy from pre-menopausal (aged 34–50), self-identified Caucasian and African American women. No fibroids were detected by ultrasound prior to surgery in the non-fibroid patients. All patients who participated to the study gave consent to donate tissue for this study through the Spectrum Health Biorepository. Tissues were washed with phosphate-buffered saline, dissected, and chopped into smaller pieces (4–9 mm^2^), some of which were immersed in RNAlater (Sigma, Saint Louis, MO) and stored at 4 °C for qPCR and RNA-seq analyses. For long-term storage, samples were placed at −20 °C after an overnight incubation in RNAlater. The remaining tissue pieces were immediately flash frozen and stored at −80 °C for subsequent protein isolation or other experiments. *MED12* mutation in the fibroids was determined by PCR amplification followed by Sanger sequencing using primers 5′-CTTCGGGATCTTGAGCTACG-3′ and 5′-GGAGGGTTCCGTGTAGAACA-3′ for Exon1, primers 5′-GCTGGGAATCCTAGTGACCA-3′ and 5′-GGCAAACTCAGCCACTTAGG-3′ targeting Exon 2. *MED12* cDNA was amplified using primers 5′-CTTCGGGATCTTGAGCTACG-3′ and 5′-AAGCTGACGTTCTTGGCACT-3′ spanning Exon 1 and Exon 2.

### 4.2. RNA Isolation

Total RNA was isolated from frozen tissues stored in RNAlater. Tissues were homogenized in TRIzol reagent (Thermo Fisher Scientific, Fairlawn, NJ, USA) and RNA was isolated following the manufacturer’s instructions. Isolated RNA was stored at −80 °C in nuclease-free water. Nanodrop 1000 spectrophotometer (Thermo Fisher Scientific, Fairlawn, NJ, USA) and Agilent 2100 Bioanalyzer (Agilent Technologies, Santa Clara, CA, USA) instruments were used to measure RNA concentration and quality, according to the manufacturers’ protocols. RNA integrity values of ≥7.5 were required for further processing.

### 4.3. Library Preparation and Sequencing

High quality RNA samples (*n* = 6/group) were submitted to the Van Andel Research Institute (VARI) Genomics Core for library preparation and paired-end (2 × 75 bp) RNA-sequencing on an Illumina NextSeq 500 instrument (Illumina Inc., San Diego, CA, USA). Libraries were prepared using a Kapa RNA HyperPrep kit with ribosomal reduction, pooled, and sequenced on flowcells to yield approximately 50–60 million reads/sample. Reads were trimmed for quality and adapters using TrimGalore (version 0.6.5) [51] and quality trimmed reads were assessed with FastQC (version 0.11.7). Trimmed reads were mapped to *Homo sapiens* (human) genome assembly GRCh38 (hg38) using STAR (version 2.6.1c) [52]. Reads overlapping Ensembl annotations (version 99) were quantified with STAR prior to model-based differential expression (DE) analysis using the edgeR-robust method [52]. Genes with low counts per million (CPM) were removed using the filterByExpr function from edgeR.

### 4.4. RNA-Seq Analyses

A scatterplot of the first two principal components was constructed with the plotMDS function of edgeR to verify sample separation prior to statistical testing. A 3D principal component plot was generated for all three groups using the pca3D package (version 0.10.2). Genes were considered differentially expressed if their respective edgeR-robust false discovery rates (FDR) corrected *p*-values were less than 0.05. Differential expression was calculated by comparing MF versus M, F versus M, or F versus MF. Differentially expressed genes (DEGs) were visualized with volcano plots and heatmaps generated using the EnhancedVolcano (version 1.8.0) and pheatmap (version 1.0.12) packages in R. Raw FASTQ files were deposited in the NCBI Gene Expression Omnibus (GSE169255). Downstream analyses of RNA-seq results were completed using the clusterProfiler (version 3.16.1) [53] package in R with an FDR *p*-value cutoff of 0.05. Gene set enrichment analyses were conducted with all expressed genes using the 50 Hallmark gene sets collection (H) [24] downloaded from the Molecular Signatures Database (MSigDB) [54,55]. Disease Ontology (DO) gene sets were used to identify over-represented diseases from DEGs with the DOSE R package (version 2.3.5) [25]. Kyoto Encyclopedia of Genes and Genomes (KEGG) pathway of WNT (hsa04310) and MAPK pathways (hsa04010) were generated using pathview (version 1.28.1). Gene Ontology (GO) GSEA was used to find biological process (BP) down or upregulated between comparisons. The top enriched GSEA terms were shown in the figures. Venn diagrams were constructed to visualize overlapping genes between groups or gene sets using the venn package (version 1.9). The function category netplot function (cnetplot) from ClusterProiler was used to associate identified GO BPs with genes that may belong to multiple annotation categories.

### 4.5. Quantitative Real Time PCR

cDNA was synthesized using the SuperScript IV Reverse Transcriptase kit (Invitrogen, Carlsbad, CA, USA) with 1 μg of the total RNA input for confirmation of the RNA-seq results. Quantitative Real time PCR (qRT-PCR) analysis using SYBRGreen (BioRad, Hercules, CA, USA) was performed to determine relative gene expression using the ViiA 7 qRT-PCR System (Applied Biosystems, Foster City, CA, USA). RPL17 was used as a reference gene for data normalization. Primer sequences used for qRT-PCR (5′-3′) were as follows; *RPL17* forward (ACGAAAAGCCACGAAGTATCTG), *RPL17* reverse (GACCTTGTGTCCAGCCCCAT), *CCND1* forward (AGCTCCTGTGCTGCGAAGTGGAAAC), *CCND1* reverse (AGTGTTCAATGAAATCGTGCGGGGT).

### 4.6. Western Blot

Proteins were extracted from flash frozen tissues, using RIPA lysis buffer containing a protease inhibitor cocktail (0.1 ug/mL each pepstatin A, chymostatin, antipain A, leupeptin, 1 ug/mL, aprotinin, and 0.1 mM phenylmethylsulfonyl fluoride). Equal amounts of total protein (25 μg) were resolved via 4–12% (wt/vol) polyacrylamide, Bis-Tris gradient gels (Thermo Fisher Scientific, Fairlawn, NJ, USA) and transferred onto nitrocellulose membranes. Membranes were blocked with 5% (wt/vol) nonfat dry milk for 1 h at room temperature (RT) and incubated overnight at 4 °C with 0.2 μg/mL anti-CCND1 (RB-9041-P1; Thermo Scientific, Fairlawn, NJ, USA) in 5% BSA. Immunoreactive proteins were visualized on ChemiDoc (Bio-Rad, Hercules, CA, USA) following incubation with pre-adsorbed Fab fragments of horseradish peroxidase-linked antirabbit secondary antibodies (1:10,000; Jackson Immunoresearch, West Grove, PA, USA) for 1 h at RT, developed with ECL reagent (Cytiva, Marlborough, MA, USA). β-tubulin (TUBB, 1:5000 in 5% nonfat dry milk for 1 h at RT; T5201; Sigma, Saint Louis, MO, USA) was used as a loading control. The band intensity was quantified using Image lab (Version 5.1, Bio-Rad), and normalized to corresponding β- tubulin bands.

### 4.7. Statistical Analyses

Bioinformatic statistics were performed using the listed packages in R (version 4.0.4). Differentially expressed genes were identified as those having an FDR corrected *p*-value < 0.05. Data with unequal variances were log transformed, and homogeneity of variances verified before completion of analyses. The Benjamini–Hochberg procedure was used to control FDR for gene ontology (GO) enrichment analyses. Gene expression was measured in triplicate by qRT-PCR, fold-changes were calculated by the ΔΔCt method, and analyzed with Prism (version 9.0.2, GraphPad, San Diego, CA, USA).

## Data Availability

Raw FASTQ files were deposited in the NCBI Gene Expression Omnibus (GSE169255).

## Supplementary Materials

The following are available online at https://www.mdpi.com/article/10.3390/ijms22073618/s1, Figure S1: Comparison with JW George et al. 2019 DEGs, Figure S2: Boxplots of genes overlapping with the leiomyoma disease ontology shown in Figure 4E, Figure S3: Visualization of KEGG WNT Signaling Pathway, Figure S4: Visualization of KEGG MAPK Pathway, Supplementary File 1: Differentially Expressed Genes of Myometrial Samples from Fibroid Patients vs Myometrial Samples from Non-Fibroid Patients and Supplementary File 2: Differentially Expressed Genes of Fibroid Samples vs Myometrial Samples from Non-Fibroid Patients.
