# Supplementary material for: Transcriptome Analyses of Myometrium from Fibroid Patients Reveals Phenotypic Differences Compared to Non-Diseased Myometrium
Source: Int J Mol Sci. 2021 Mar 31;22(7):3618. doi: 10.3390/ijms22073618 (PMC8036618; doi:10.3390/ijms22073618)
Supplement: Supplementary file 1 [file ijms-22-03618-s001.zip › Figure S3-1.pdf]

Figure S3

A

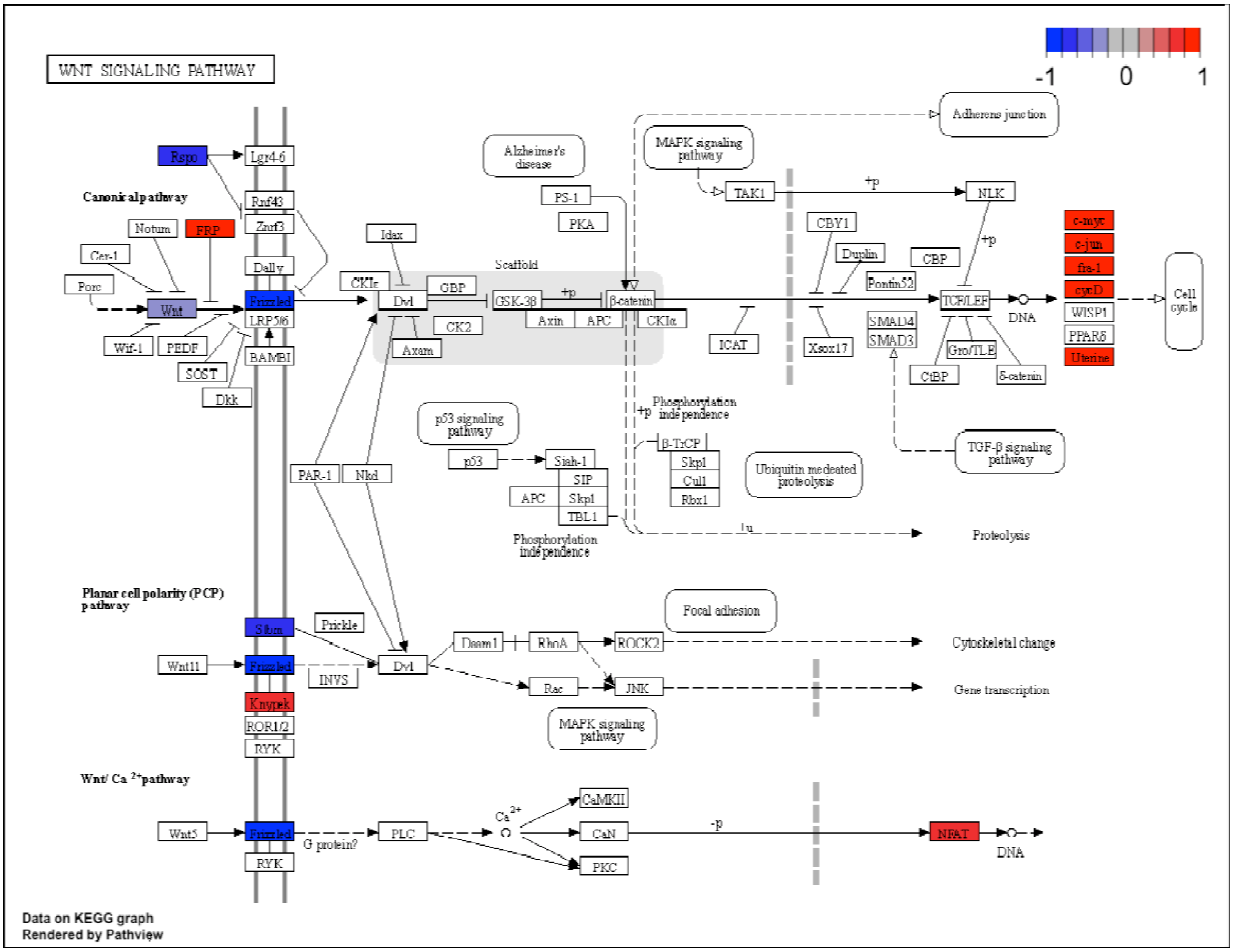

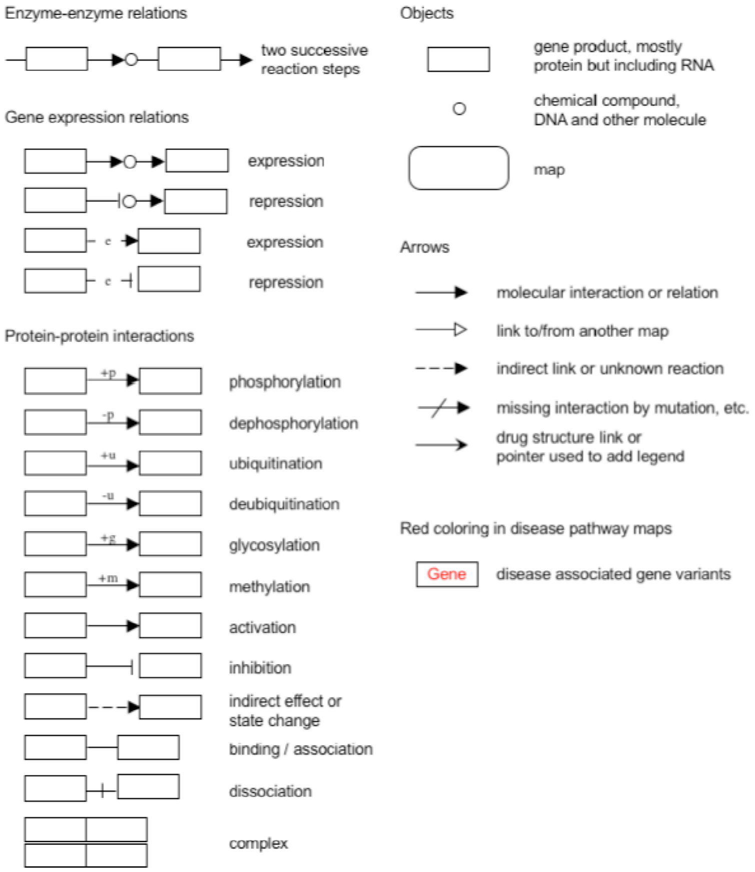

Figure S3. Visualization of KEGG WNT signaling pathway

Expression profiles of WNT signaling (hsa04310) related genes visualized on KEGG pathway diagram. Red and blue indicate upregulated or downregulated genes respectively. MF vs M (A), F vs M (B), and F vs MF (C).
