# Supplementary material for: Transcriptome Analyses of Myometrium from Fibroid Patients Reveals Phenotypic Differences Compared to Non-Diseased Myometrium
Source: Int J Mol Sci. 2021 Mar 31;22(7):3618. doi: 10.3390/ijms22073618 (PMC8036618; doi:10.3390/ijms22073618)
Supplement: Supplementary file 1 [file ijms-22-03618-s001.zip › Figure S4.pdf]

A

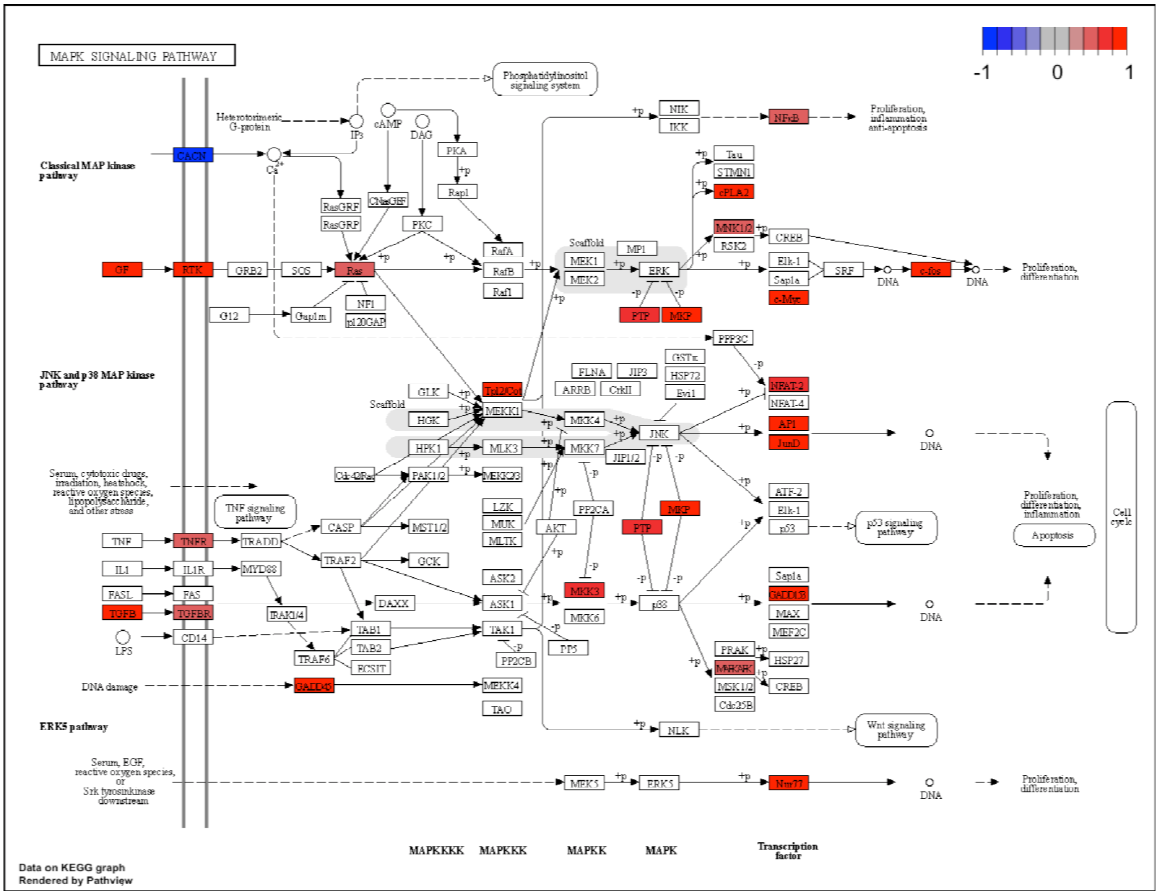

B

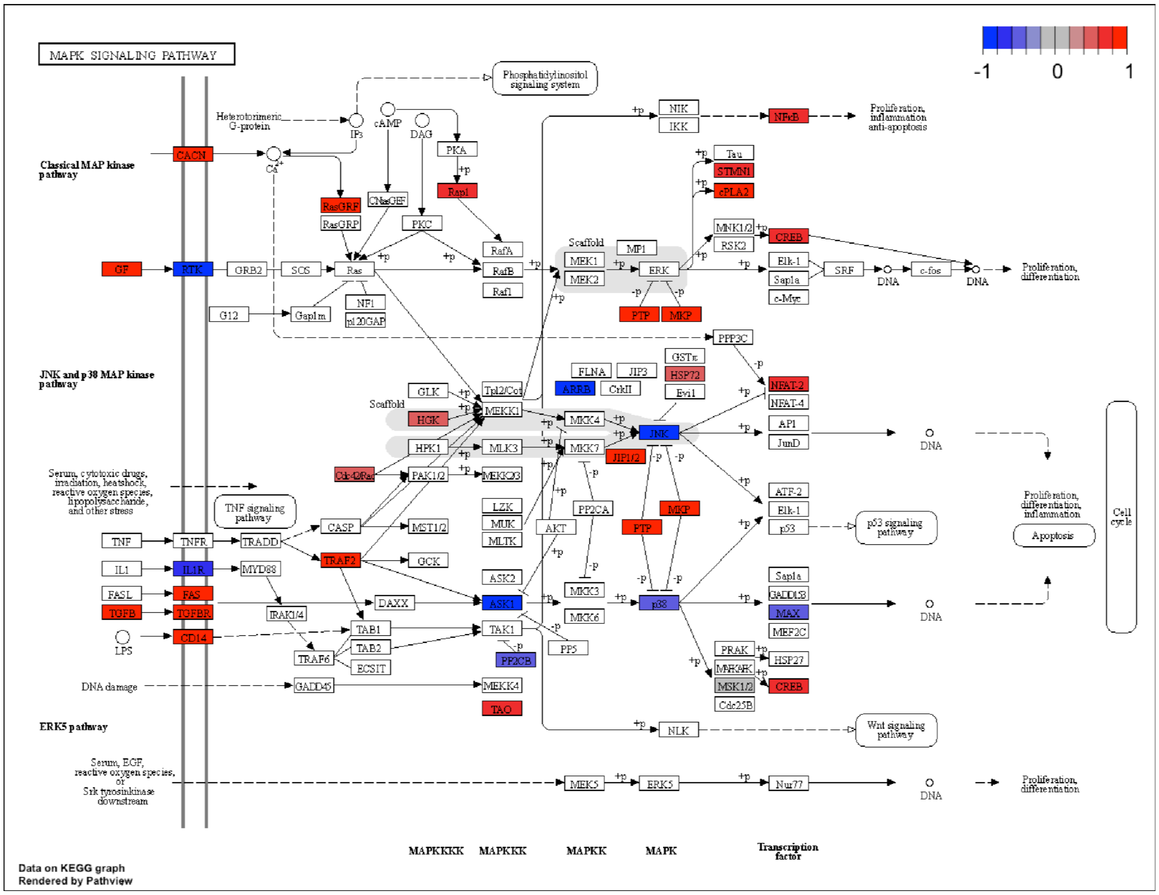

Figure S4. Visualization of KEGG MAPK pathway

Expression profiles of MAPK (hsa04010) related genes visualized on KEGG pathway diagram. Red and blue indicate upregulated or downregulated genes respectively. MF vs M (A), and F vs M (B).
