# Supplementary material for: Transcriptome Analyses of Myometrium from Fibroid Patients Reveals Phenotypic Differences Compared to Non-Diseased Myometrium
Source: Int J Mol Sci. 2021 Mar 31;22(7):3618. doi: 10.3390/ijms22073618 (PMC8036618; doi:10.3390/ijms22073618)
Supplement: Supplementary file 1 [file ijms-22-03618-s001.zip › Figure S1.pdf]

Figure S1

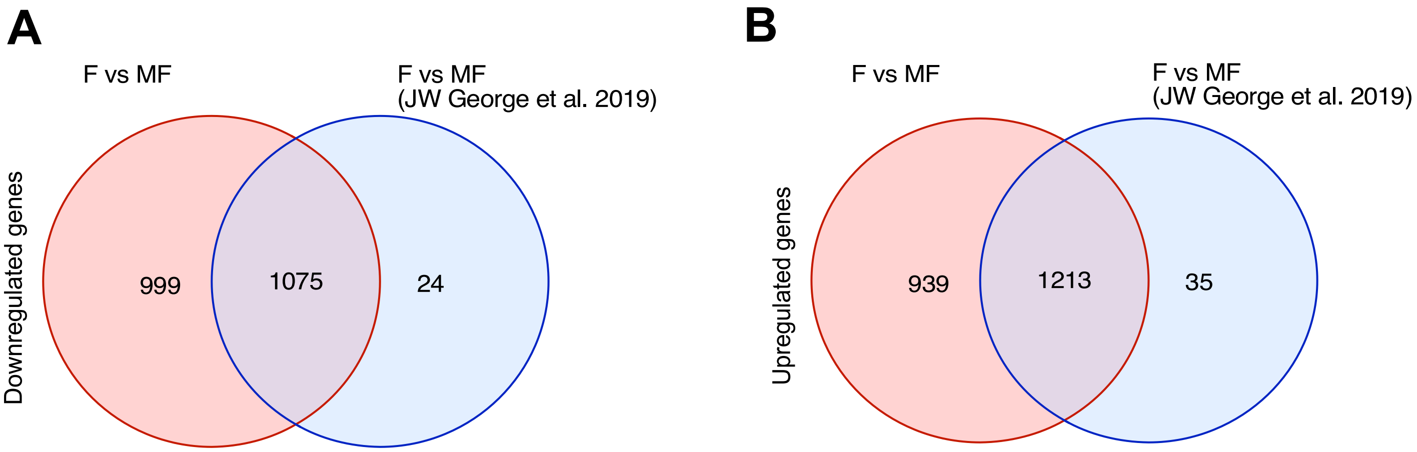

Figure S1. Comparison with JW George et al. 2019 DEGs

Venn diagrams illustrate the overlap for downregulated (A) and upregulated genes (B) between F vs MF and F vs MF from a previous study.
