# Supplementary material for: Transcriptome Analyses of Myometrium from Fibroid Patients Reveals Phenotypic Differences Compared to Non-Diseased Myometrium
Source: Int J Mol Sci. 2021 Mar 31;22(7):3618. doi: 10.3390/ijms22073618 (PMC8036618; doi:10.3390/ijms22073618)
Supplement: Supplementary file 1 [file ijms-22-03618-s001.zip › Figure S2.pdf]

Figure S2

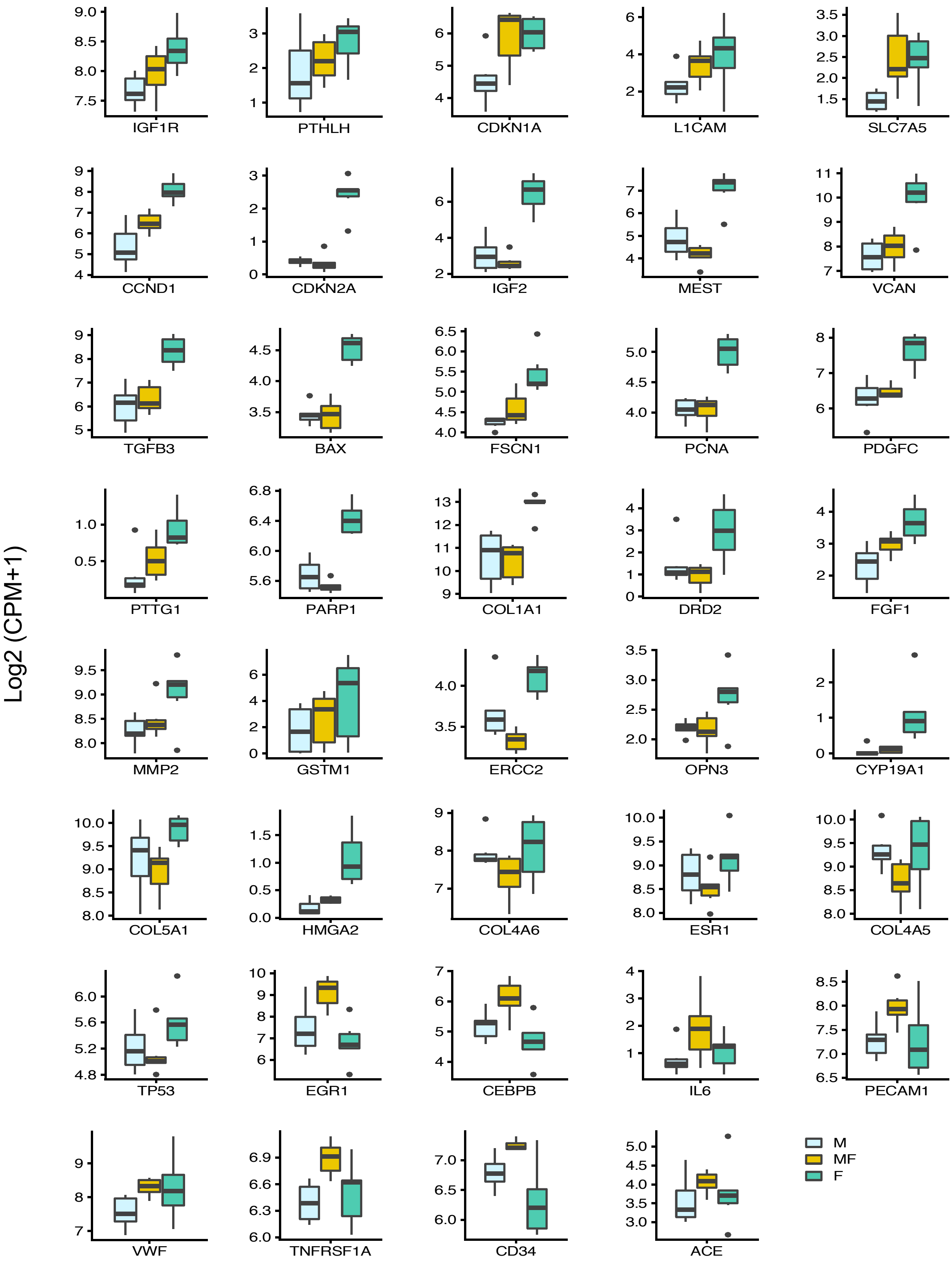

Figure S2. Boxplots of genes overlapping with the leiomyoma disease ontology shown in Fig 4E

Significant genes overlapping with leiomyoma Disease Ontology gene set and (A) F vs M-specific, (B) shared F vs M and MF vs M, (C) shared with the 3 comparisons, (D) shared F vs M and F vs MF, (E) F vs MF-specific, (F) MF vs M-specific. Gene expression is represented as Log_2(CPM +1).
