# Supplementary figures and images for: Transcriptome Analyses of Myometrium from Fibroid Patients Reveals Phenotypic Differences Compared to Non-Diseased Myometrium
Source: Int J Mol Sci. 2021 Mar 31;22(7):3618. doi: 10.3390/ijms22073618 (PMC8036618; doi:10.3390/ijms22073618)

B

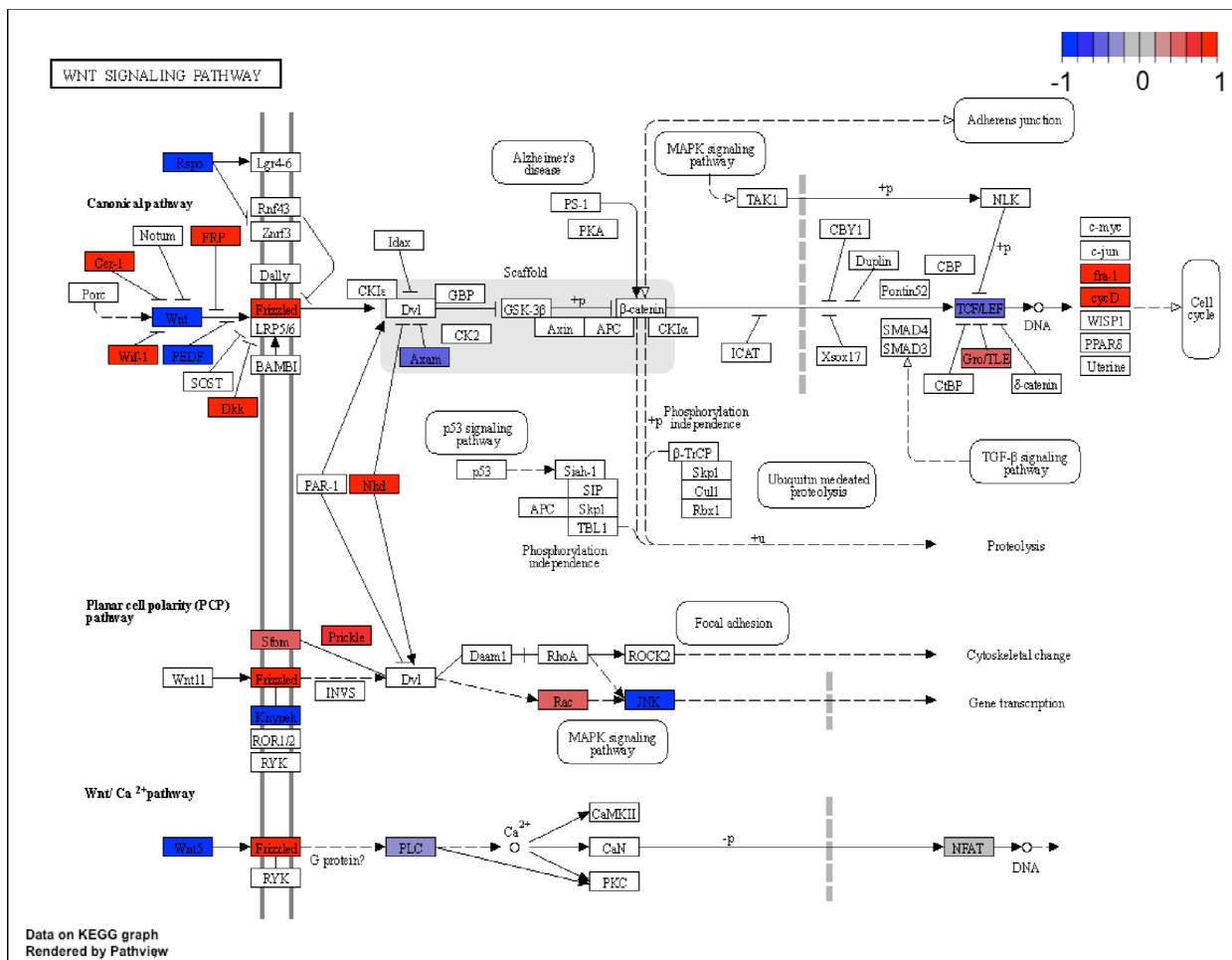

C

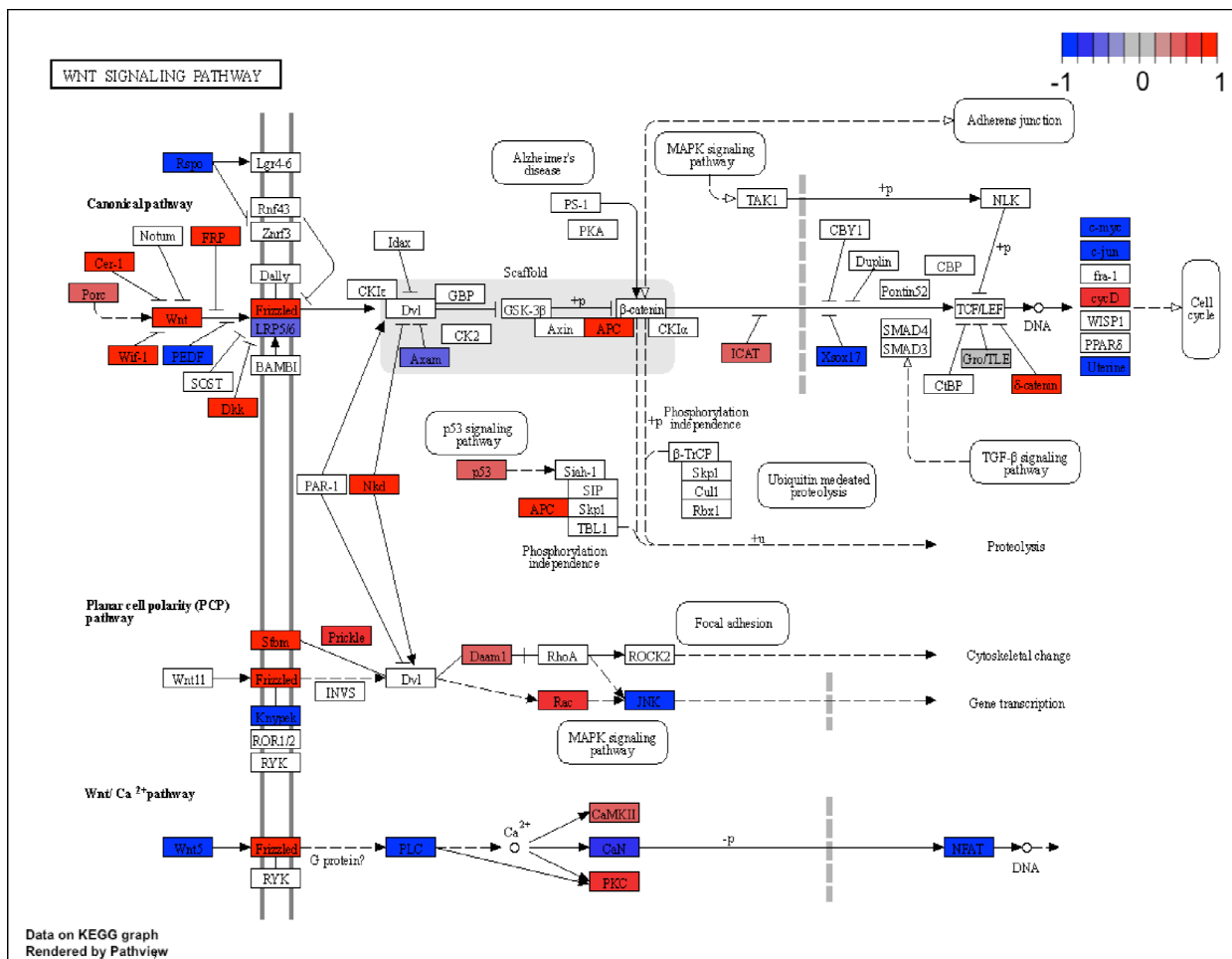

Supplement: Supplementary file 1 [file ijms-22-03618-s001.zip › Figure S3-2.pdf]
